# Whole Blood Profiling of Bacillus Calmette–Guérin-Induced Trained Innate Immunity in Infants Identifies Epidermal Growth Factor, IL-6, Platelet-Derived Growth Factor-AB/BB, and Natural Killer Cell Activation

**DOI:** 10.3389/fimmu.2017.00644

**Published:** 2017-06-06

**Authors:** Steven G. Smith, Johanneke Kleinnijenhuis, Mihai G. Netea, Hazel M. Dockrell

**Affiliations:** ^1^Faculty of Infectious and Tropical Diseases, Department of Immunology and Infection, London School of Hygiene and Tropical Medicine, London, United Kingdom; ^2^Department of Internal Medicine, Radboud Center for Infectious Diseases, Radboud University Medical Center, Nijmegen, Netherlands

**Keywords:** bacillus Calmette–Guérin, vaccination, heterologous effects, trained immunity, infants, cytokines, chemokines, natural killer

## Abstract

Vaccination of infants with bacillus Calmette–Guérin (BCG) activates both the innate and adaptive arms of the immune response. The antimycobacterial effects of these responses most likely account for the ability of BCG to protect against childhood forms of tuberculosis (TB). There is also evidence for a heterologous protective effect of BCG vaccination against TB-unrelated mortality in low birth weight infants. A possible mechanism of action of this effect, the induction of trained innate immunity, has been demonstrated when cells from BCG-vaccinated adults are restimulated *in vitro* with non-related microbial stimuli. Our aim was to examine an extensive panel of secreted immune biomarkers to characterize the profile of trained innate immunity in infants. Stimulation of whole blood for 48 h was performed 4 months after BCG vaccination, or in control unvaccinated infants. Stimulants were lipopolysaccharide; Pam3Cys (P3C); heat-killed *Candida albicans, Staphylococcus aureus, Escherichia coli*, and a lysate of *Mycobacterium tuberculosis*. Culture supernatants were tested for secreted cytokines and chemokines by 42-plex bead array and monocytes and natural killer (NK) cells assessed for expression of activation markers by flow cytometry. BCG-vaccinated infants displayed increases in 11 cytokines and chemokines in response to different non-specific innate immunity stimuli: epidermal growth factor (EGF); eotaxin; IL-6; IL-7; IL-8; IL-10; IL-12p40; monocyte chemotactic protein-3; macrophage inflammatory protein-1α; soluble CD40 ligand and platelet-derived growth factor (PDGF)-AB/BB. Although each stimulant induced a distinct response profile, three analytes, EGF, IL-6, and PDGF-AB/BB, were commonly higher after stimulation with Pam3Cys, *C. albicans*, and *S. aureus*. Conversely, certain cytokines such as interferon gamma-inducible protein-10, IL-2, IL-13, IL-17, GM-CSF, and GRO were suppressed in BCG-vaccinated infants, while no increases in TNFα or IL-1β production were detected. We did not observe a concomitant, BCG-associated change in monocyte surface activation markers in response to non-specific stimuli, but we detected a significant increase in CD69 expression on NK cells in response to Pam3Cys. Pam3Cys-induced NK cell activation correlated with the magnitude of IL-12p40 and IL-10 responses to the same stimulant. This study reveals a novel cytokine/chemokine biomarker signature of BCG-induced trained innate immunity in infants and the involvement of NK cells in these responses.

## Introduction

*Mycobacterium bovis* bacillus Calmette–Guérin (BCG) is currently the only licensed vaccine for tuberculosis (TB). Although its protective efficacy against adult pulmonary TB is variable (1–3), BCG affords more reliable protection against childhood forms of the disease when administered to infants (4). The immune mechanisms responsible for this protection are not fully understood, although many studies have described BCG-induced, antigen-specific immune responses that may play a part (5–12).

Evidence also exists of a beneficial effect for the BCG vaccine against several different diseases and outcomes other than TB, including as a therapeutic agent against bladder cancer when instilled directly into the bladder (13) and protection against all-cause mortality in low birth weight infants (14). These effects are heterologous (non-specific) as they do not rely on mycobacteria-specific adaptive immune responses. Exactly how BCG mediates these effects is not clear, although different pathways are probably involved. Non-specific protective effects against mortality in low birth weight infants are a result of resistance to pathogenic microorganisms that are responsible for death due to neonatal sepsis and respiratory infections (15, 16). The immune responses mediating this effect could be either heterologous T-cells (i.e., T-cells induced by an epitope from one organism but with cross-reactivity to others) or the ability of BCG to potentiate the responsiveness of the innate immune system to later infections: a biological process termed trained innate immunity which represents a *de facto* innate immune memory (17–19). Such a phenomenon is thought to have existed for millions of years as acquired systemic resistance in plants and also in invertebrates, neither of which have adaptive immune systems (17). Several studies have revealed enhancements of the neonatal innate immune response to Gram-positive and Gram-negative infections following previous encounters, mediated by different toll-like receptor pathways (20) or initiated by *in utero* inflammatory conditions such as histologic chorioamnionitis (21). Studies in adults show that trained innate cells, including monocytes and natural killer (NK) cells, appear to be epigenetically and metabolically reprogrammed to produce increased amounts of pro-inflammatory cytokines and display higher levels of surface activation markers in response to restimulation with toll-like receptor ligands or different whole microorganisms (22–25).

If trained immunity is to provide a mechanism of action for the non-specific protective effects of BCG, evidence of trained immunity in infants or infant innate cells is needed. The immune system of the newborn infant differs from that of the adult in its constitution as well as in its propensity to respond to different stimuli; differences that reflect the unique physiological challenge of transitioning from the intrauterine environment to the outside world where some microorganisms are beneficial commensals and some are life-threatening pathogens (26, 27). Newborn innate immune responses are characterized by a reduced capacity for pro-inflammatory cytokines and dendritic cell differentiation and activation, but a greater propensity to produce regulatory cytokines (28, 29). More data are needed on how these differences impact upon the generation of trained innate immunity by BCG.

Our aim in this study was to probe the infant immune response to mycobacteria-unrelated stimuli following BCG vaccination for potential mediators of trained immunity. We used a multiplex bead array approach for the detection of secreted cytokines and chemokines from diluted whole blood following stimulation with a panel of innate stimuli. The rationale for this was to maximize the variety of immune cells available for stimulation as well as the potential to detect a broad array of soluble mediators. Our data provide a description of previously unreported profiles of trained immunity in infants and a role for activated NK cells.

## Materials and Methods

### Study Participants and Sample Collection

Healthy, UK-born infants were recruited following ethical approval from the National Research Ethics Service Committee London-East (11/LO/0363) and from the Ethics Committee of the London School of Hygiene and Tropical Medicine (ref. 4068). Written consent was obtained from parents prior to recruitment. Infants were recruited from two regions of South East England: Redbridge, where a single dose of intradermal BCG (BCG Vaccine Danish Strain 1331, Staten Serum Institute, Copenhagen, Denmark) was administered to infants at approximately 6 weeks of age in local vaccination clinics and West Essex where infants do not routinely receive BCG. Heparinized venous blood was obtained 4 months post-vaccination or from unvaccinated infants at an age-matched time point. This exploratory study was part of a larger study (11) and 4-month post-vaccination samples available were *n* = 11, vaccinated infants; and *n* = 10, unvaccinated infants for Luminex analysis and *n* = 10 and *n* = 8, respectively, for flow cytometry analysis.

### Diluted Whole Blood Assays for Cytokine Responses to Innate Stimuli

Venous blood was diluted 1/5 in RPMI 1640 (Invitrogen) supplemented with 2 mM l-glutamine (Invitrogen) and cultured at 37°C for 48 h in 96-well U-bottomed plates in a final volume of 200 µl. Duplicate wells were incubated alone (medium only negative control) or with the following stimuli: lipopolysaccharide (LPS; 10 ng/ml); (S)-(2,3-bis(palmitoyloxy)-(2RS) -propyl)-*N*-palmitoyl-(R)-Cys-(S)-Ser(S)-Lys_4_-OH, trihydrochloride [Pam3Cys; 10 µg/ml]; heat-killed (HK) *Candida albicans* (*C. albicans*; 10^6^ microorganisms/ml); HK *Staphylococcus aureus* (*S. aureus*; 10^6^ microorganisms/ml); HK *Escherichia coli* (*E. coli*; 10^6^ microorganisms/ml); and sonicated *Mycobacterium tuberculosis* H37Rv [*Mtb* lysate; 1 µg/ml end concentration]. Concentrations used for each stimulus were optimized in previous experiments.

### Tissue Culture Supernatant Harvest and Multiplex Bead Array Assay

After 48 h, plates were centrifuged at 400 *g* for 5 min. Supernatants were removed from duplicate wells, pooled, and stored in aliquots at −80°C prior to analysis. Thawed supernatants were subjected to multiplex bead array analysis using the human cytokine/chemokine Milliplex™ MAP 42-plex pre-mixed kit (Merck Millipore) and following the manufacturer’s instructions. The pre-mixed bead set included the following panel: IL-1α, IL-1β, IL-1Ra, IL-2, IL-3, IL-4, IL-5, IL-6, IL-7, IL-8, IL-9, IL-10, IL-12p40, IL-12p70, IL-13, IL-15, IL-17, epidermal growth factor (EGF), eotaxin, Flt-3L, FGF-2, fractalkine, G-CSF, GM-CSF, GRO, IFNα2, IFNγ, IP-10, MCP-1, MCP-3, MDC, MIP-1α, MIP-1β, platelet-derived growth factor (PDGF)-AA, PDGF-AB/BB, RANTES, sCD40L, sIL-2Ra, TGFα, TNFα, TNFβ, and VEGF. Data were acquired using the Biorad Luminex^®^ 100 system and Bioplex Manager Software version 6.1 (Biorad).

### Peripheral Blood Leukocyte (PBL) Harvest and Flow Cytometry

Following the removal of supernatants from diluted whole blood assay plates, remaining PBLs were harvested and cryopreserved for later analysis. EDTA in PBS (2 mM) was added to assay wells, which were incubated at room temperature for 15 min to detach adherent cells. Wells were mixed and cells from duplicate wells were pooled and then incubated with 10× volume of 1× FACS Lysing Buffer (BD Biosciences) at room temperature for 10 min. Following red cell lysis, PBLs were pelleted, resuspended in fetal bovine serum with 10% dimethylsulfoxide, and cryopreserved in liquid nitrogen. For flow cytometric analysis, thawed cells were washed in PBS with 0.1% bovine serum albumin and 0.01% sodium azide (both Sigma Aldrich) and stained for 30 min at 4°C with the following antibodies: CD3-BV510 (clone UCHT1); CD25-PerCP-Cy™5.5 (clone M-A251); CD56-PE-Cy™7 (clone B159); CD206-PE-CF594 (clone 19.2); HLA-DR-BV605 (clone G46-6) (all from BD Biosciences); CD11b-FITC (clone ICRF44); CD69-PE (clone FN50); CD163-APC (clone eBioGHI/61) (all from Affymetrix/eBiosciences); CD14-BV650 (clone M5E2; Biolegend). Following a further wash, cells were resuspended in PBS with 1% paraformaldehyde for acquisition. Cells were acquired using a BD LSR II flow cytometer (BD Biosciences) equipped with blue (488 nm), red (633 nm), and violet (405 nm) lasers and FACSDiva 6.1.3 software. Compensation was carried out using BD™ CompBead Plus compensation particles stained separately with each antibody conjugate and fluorescence minus one control stains were used to determine the position of phenotype and activation marker analysis gates.

### Data Analysis, Management, and Statistical Analysis

Multiplex bead array data for each stimulation condition were background subtracted using values measured in unstimulated controls. Background levels of each cytokine/chemokine are given in Table S1 in Supplementary Material. Final concentrations below the lower limit of quantitation were adjusted to the value of the lowest standard (3.2 pg/ml) and data above the upper limit of quantitation were adjusted to the highest standard (10,000 pg/ml). The compensation matrix was generated and flow cytometric data were analyzed using FlowJo™ 10.2 software (FlowJo LLC). Monocytes were gated based on CD14 and HLA-DR expression as well as on size and granularity. NK cells were gated based on CD56 expression and lack of CD3 expression (see Figure S1 in Supplementary Material). Multiplex bead array data were analyzed using IBM^®^ SPSS^®^ Statistics version 23 software (IBM Corp.) and Prism 7 for Windows (GraphPad Software Inc.). Flow cytometric data were further analyzed using a combination of Spice 5.35 (30) and Prism. Statistical comparisons between vaccinated and unvaccinated infant groups for both multiplex bead array and flow cytometry data were by Mann–Whitney *U* test and correlations were by Spearman’s rank correlation coefficient.

## Results

### Infant BCG Vaccination Is Associated with Enhanced Cytokine and Chemokine Responses to Non-Specific Innate Stimuli

Diluted whole blood from BCG-vaccinated and unvaccinated infants was stimulated with a panel of non-specific innate stimuli and with a preparation of *Mtb* lysate. Secreted cytokines and chemokines were measured in tissue culture supernatants by multiplex bead array after 48 h.

**Figure 1:**
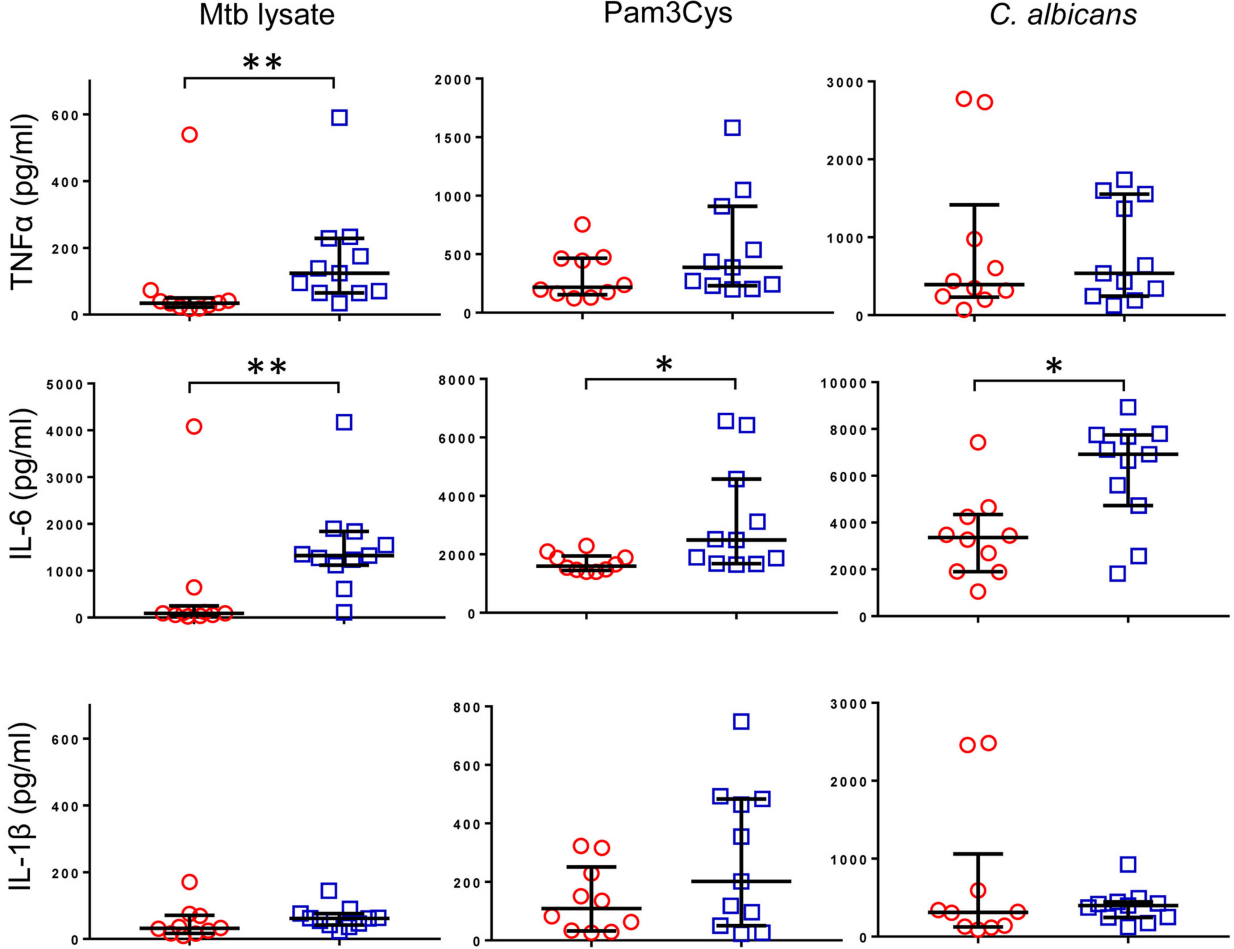
Cytokine profile of bacillus Calmette–Guérin (BCG)-induced trained immunity in infants does not include TNFα or IL-1β. TNFα, IL-6, and IL-1β concentrations were measured in tissue culture supernatants after diluted whole blood from BCG-vaccinated (□, *n* = 11) and unvaccinated (○, *n* = 10) infants was cultured for 48 h with the indicated stimulants. Data shown is background subtracted (unstimulated samples). Wide bars indicate median response; narrow bars indicate 25th and 75th percentiles. Responses in vaccinated and unvaccinated groups were compared using the Mann–Whitney *U* test: **p < 0.01; *p < 0.05.

**Figure 2:**
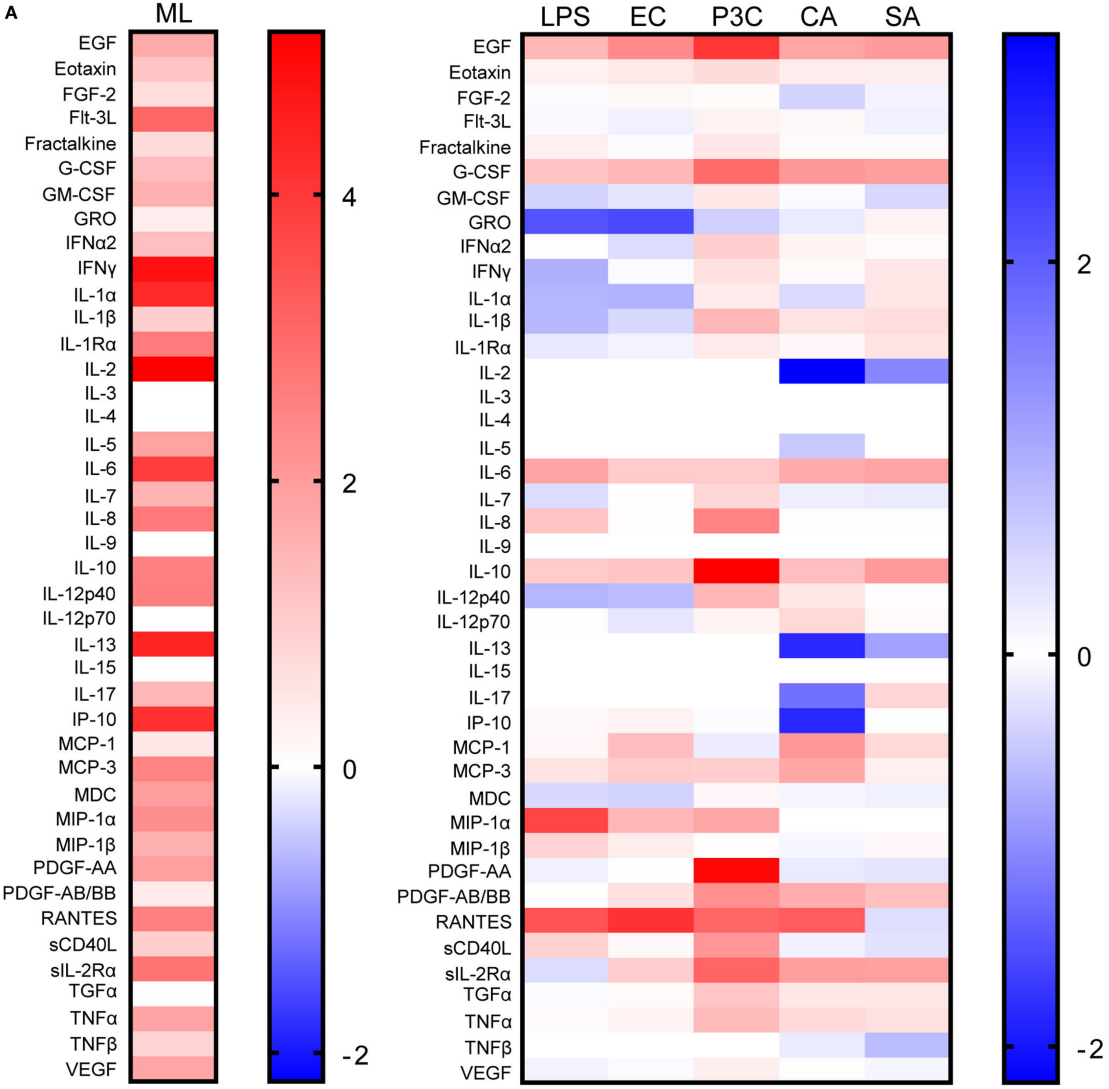

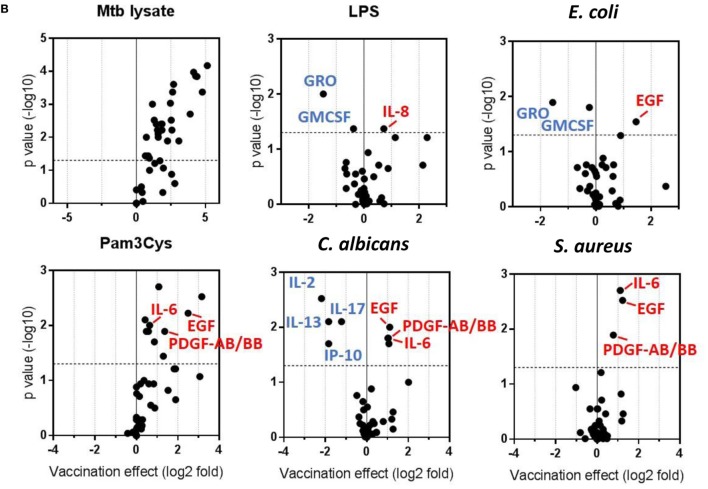
Distinct patterns of cytokine and chemokine secretion characterize bacillus Calmette–Guérin (BCG)-induced trained immunity in response to different, non-specific stimuli. The concentrations of 42 cytokines and chemokines were measured in 48 h, diluted whole blood assay supernatants using multiplex bead array. **(A)** Heat maps represent the vaccination effect [the log2 median fold change in analyte concentration in the BCG-vaccinated infants (*n* = 11) compared to unvaccinated infants (*n* = 10)] on the indicated cytokine/chemokine responses to different stimuli: ML, *Mtb* lysate; LPS, lipopolysaccharide; EC, *E. coli*; P3C, Pam3Cys; CA, *C. albicans*; SA, *S. aureus*. **(B)** Volcano plots represent the vaccination effect on cytokine/chemokine responses to different indicated stimuli which are plotted on the *x*-axis against the −log10 *p* value on the *y*-axis (calculated by Mann–Whitney *U* test comparisons of responses in BCG-vaccinated and unvaccinated groups). Data points represent individual cytokines and chemokines. Significant responses of interest are labeled where clarity permits. Horizontal dotted lines represent a *p* value of 0.05.

Previous reports described TNFα, IL-6, and IL-1β as the characteristic cytokines of BCG-induced trained innate immunity in adults (24, 31). However, when we investigated this signature in infants, only IL-6 was significantly increased in response to non-specific stimuli. Re-stimulation with the mycobacterial stimulant *Mtb* lystae did produce a significant increase in TNFα (Figure 1). As expected, *Mtb* lystae induced the most extensive upregulation of responses in BCG-vaccinated infants including 27 cytokines and chemokines that characterize both innate and adaptive T-cell responses (Figures 1 and 2; Table 1). We detected 11 cytokines and chemokines that were significantly increased and 6 that were significantly decreased in BCG-vaccinated infants in response to non-specific stimulants (Figure 2; Table 1). Each non-specific stimulant induced a distinct response profile. For example, increases in 10 cytokines and chemokines were detected following Pam3Cys stimulation whereas *C. albicans* stimulation revealed BCG-associated increases in four analytes: EGF, IL-6, PDGF-AB/BB, MCP-3, but decreases in four further analytes: IL-2, IL-13, IL-17, IP-10. Despite distinct response profiles for each non-specific stimulant, we observed a common signature of increases in EGF, IL-6, and PDGF-AB/BB in response to three different stimulants: Pam3Cys, *C. albicans*, and *S. aureus*.

**Table 1:** BCG vaccination-associated cytokine and chemokine responses induced by specific and non-specific stimuli in 48 h diluted whole blood assays.

Stimulation	Overexpressed in BCG-vaccinated infants	Median fold change	Under-expressed in BCG-vaccinated infants	Median fold change
*Mtb* lysate	IL-2, IFNγ, IL-13, IL-1α, IP-10, IL-6	>10		
	Flt-3L, IL-8, IL-1Rα, IL-12p40, IL-10, MCP-3	5–10		
	MIP-1α, IL-5, TNFα, VEGF, MIP-1β, GM-CSF, IL-7, IL-17, IFNα2, eotaxin	2–5		
	CD40L, TNFβ, fractalkine, FGF- 2, TGFα	1–2		

Pam3Cys	IL-10, epidermal growth factor (EGF)	5–10		
	Platelet-derived growth factor (PDGF)-AB/BB, sCD40L, MIP-1α	2–5		
	IL-12p40, IL-6, MCP-3, IL-7, eotaxin	1–2		

*Candida albicans*	EGF, IL-6, PDGF-AB/BB, MCP-3	2–5	IL-2, IL-13, IL-17, IP-10	0.2–0.5

*Staphylococcus aureus*	EGF, IL-6	2–5		
	PDGF-AB/BB	1.7		

*Escherichia coli*	EGF	2.8	GM-CSF, GRO	0.2–1.0

Lipopolysaccharide	IL-8	1.7	GM-CSF, GRO	0.2–1.0

These data led us to conclude that BCG vaccination of infants, in this setting, induces a complex reprogramming of the innate immune system to respond mainly in an enhanced fashion when exposed *in vitro* to a panel of heterogeneous, non-specific stimuli. For a few cytokines, downregulation of production was observed after vaccination. Overall, this effect is consistent with trained immunity which is known to be mediated by cells of the innate immune system.

### NK Cell CD69 Expression Is Increased in BCG-Vaccinated Infants following Pam3Cys Re-Stimulation *In Vitro*

To determine which innate immune cells were associated with altered cytokine and chemokine responses to non-specific stimuli in BCG-vaccinated infants, we stained PBLs recovered from 48 h diluted whole blood assays for markers of monocyte and NK cell phenotype and activation.

**Figure 3:**
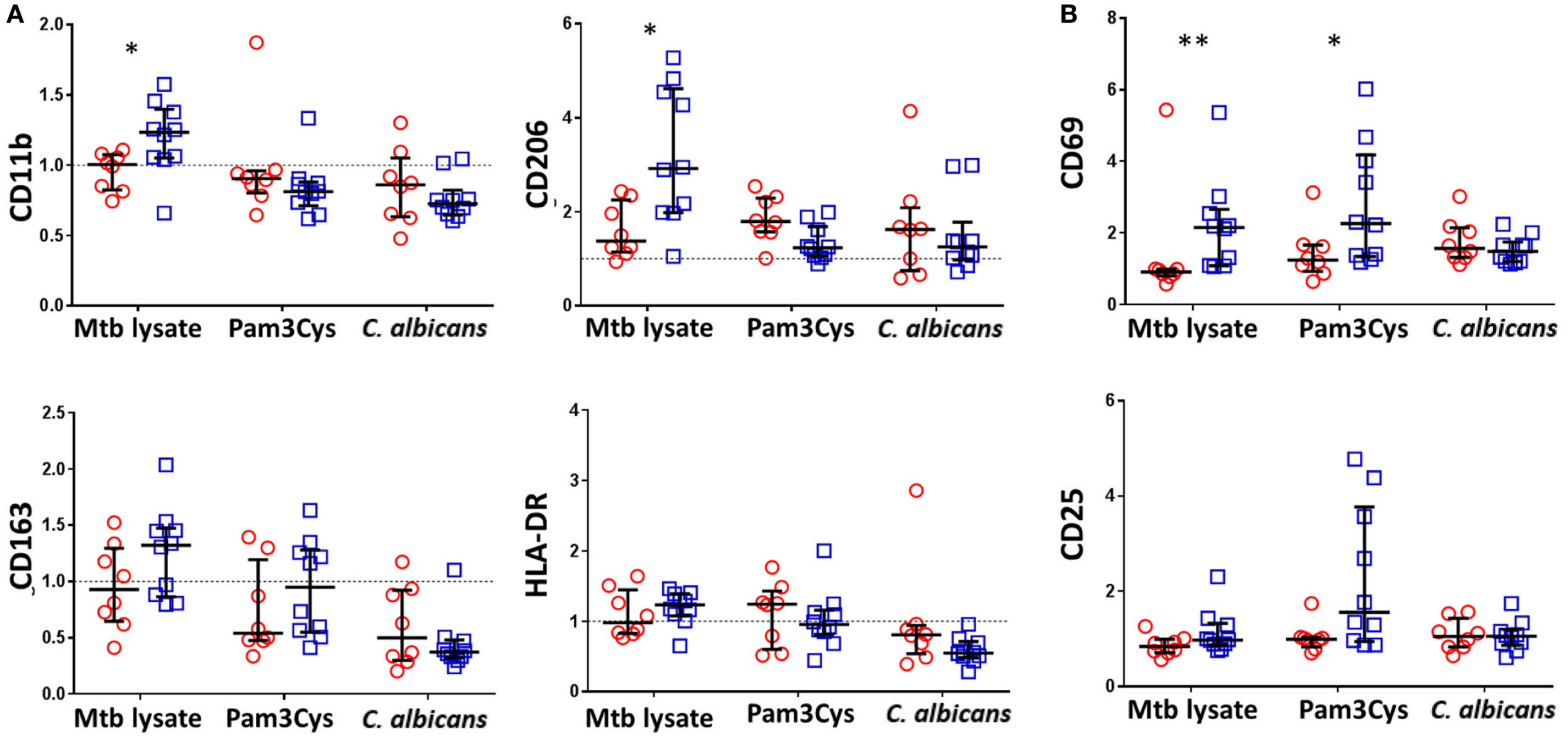
Bacillus Calmette–Guérin (BCG) vaccination of infants is associated with enhanced natural killer (NK) cell activation in response to Pam3Cys. Following 48 h, diluted whole blood assay stimulation of samples from BCG-vaccinated (□, *n* = 10) and unvaccinated (○, *n* = 8) infants, cell pellets were cryopreserved and later analyzed by flow cytometry. **(A)** Monocytes were identified based on high CD14 and HLA-DR expression as well as on size and granularity (see Figure S1A in Supplementary Material) and stained for the indicated markers of monocyte activation and differentiation. Data points represent the fold change in MFI for samples incubated with the indicated stimulants compared to unstimulated samples. **(B)** CD56+ CD3− NK cells were gated (see Figure S1B in Supplementary Material) and the expression of the activation markers CD69 and CD25 analyzed. Data points represent the fold change in percent of activation marker expressing NK cells for samples incubated with the indicated stimulants compared to unstimulated samples. Responses in vaccinated and unvaccinated groups were compared using the Mann–Whitney *U* test: *p < 0.05; **p < 0.01.

Consistent with BCG-associated increases in a broad array of cytokine and chemokine responses following *Mtb* lystae restimulation, we also detected concomitant increases in expression of CD11b and CD206 on monocytes (Figure 3A). There were no significant, BCG vaccination-associated changes to monocyte activation markers in response to non-specific stimuli. In addition to its effect on monocytes, *Mtb* lystae stimulation also induced increased surface expression of the activation marker CD69 on NK cells (Figure 3B). Although there were no significant changes to monocyte activation markers in response to non-specific stimuli, BCG-vaccinated infants displayed a significant increase in NK cell CD69 expression in response to Pam3Cys (Figure 3B).

Based on these data, we conclude that, accompanying the cytokine/chemokine profile of BCG-induced trained immunity in infants, there is a role for activated NK cells but that their involvement depends upon the nature of the restimulating non-specific ligand.

### Pam3Cys-Induced NK Cell Activation Correlates with the Secretion of IL-12p40 and IL-10

**Figure 4:**
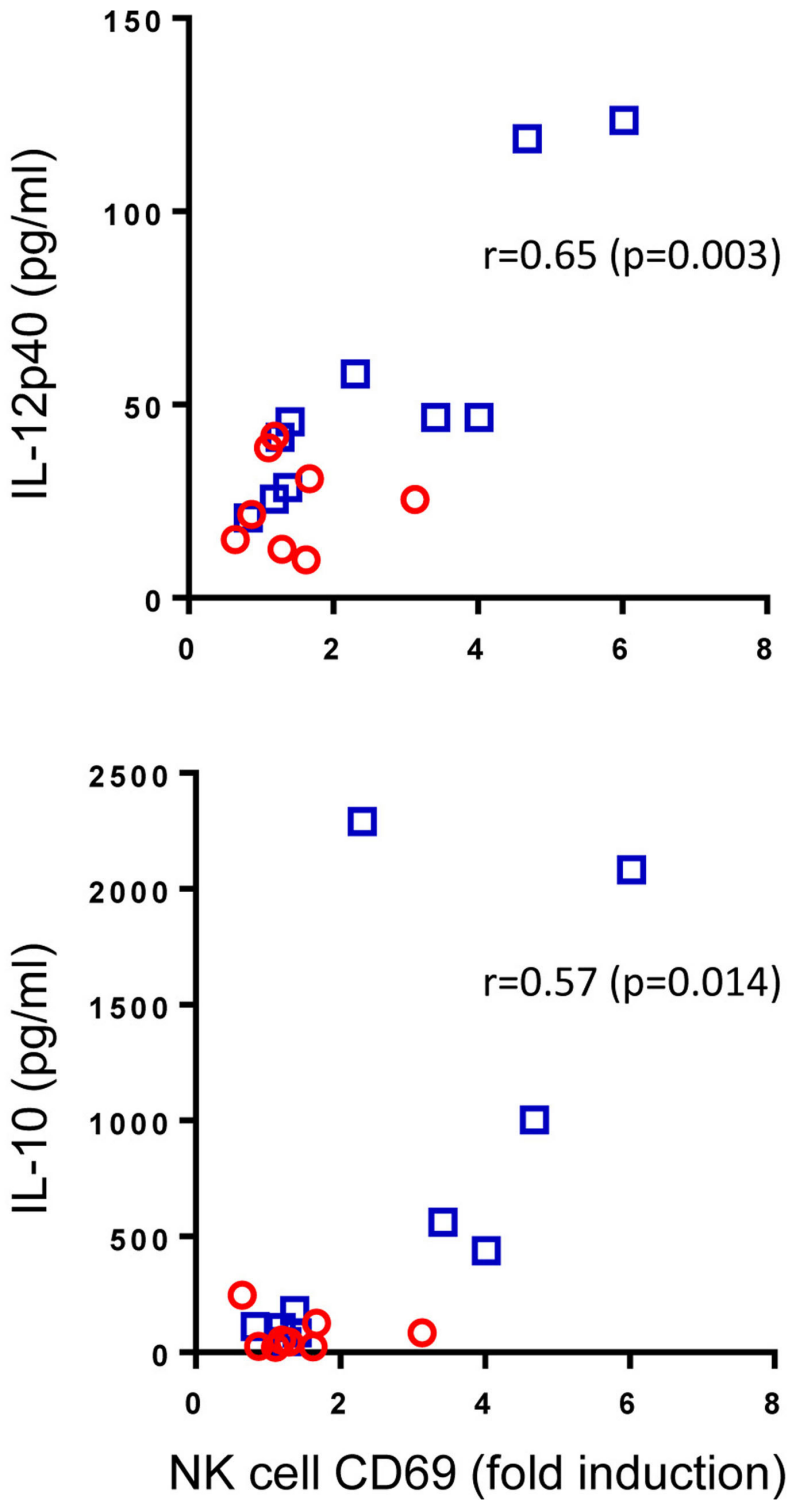
Correlation between Pam3Cys (P3C)-induced natural killer (NK) cell CD69 expression and IL-12p40 and IL-10 secretion. P3C-specific, NK cell CD69 expression in samples from bacillus Calmette–Guérin (BCG)-vaccinated (□, *n* = 10) and unvaccinated (○, *n* = 8) infants were plotted against P3C-specific secretion of IL-12p40 and IL-10 in the same samples. The degree of correlation was measured using Spearman’s rank correlation coefficient.

Pam3Cys is a ligand for TLR2 which is found on the surface of NK cells. We speculated that enhanced Pam3Cys-induced NK cell activation in BCG-vaccinated infants could be a result of changes to the cytokine milieu that this ligand induces (Figure 2) or due to intrinsic changes to NK cells following BCG vaccination that alter their responsiveness to TLR2-mediated Pam3Cys stimulation or a combination of the two. Although the scope of this study did not allow us to address the intrinsic changes hypothesis, we were able to look at associations between the magnitude of Pam3Cys-mediated NK cell activation and the changes in cytokine and chemokine release in response to the same stimulant. Of the 10 analytes that were significantly upregulated in BCG-vaccinated infants in response to Pam3Cys, IL-12p40 and IL-10 secretion demonstrated significant correlation with the extent of NK cell CD69 expression (Figure 4).

From these data, we conclude that cytokine secretion, most notably IL-12, may account at least in part for the TLR2-mediated activation of NK cells in BCG-vaccinated infants.

## Discussion

Trained innate immunity has, to date, been demonstrated and characterized largely in adults. Isolated PBMC released more TNFα, IL-6, and IL-1β when exposed to non-specific innate stimuli 3 months after BCG vaccination in adults, and monocytes displayed higher levels of activation markers (24). Mechanisms of trained immunity such as underlying epigenetic regulation and shifts in the metabolic pathways used have similarly been revealed in vaccinated adults and in adult cellular models *in vitro* (22, 32). If trained innate immunity is to provide a causal link between BCG vaccination of infants and non-specific protection against mortality due to infectious diseases other than those with a mycobacterial etiology (15, 16), then a detailed description of the phenomenon in infants and infant cells is needed.

In this paper, we have profiled the infant whole blood cytokine and chemokine secretome of trained immunity following BCG vaccination using a panel of non-specific innate stimuli and reveal a differentially expressed signature. A total of 11 analytes were overexpressed and 6 under-expressed in vaccinated infants, with different innate stimuli inducing distinct combinations of response elements: for example, the Pam3Cys-induced response included the overexpression of 10 analytes, while *S. aureus* only induced three analytes. Four analytes were overexpressed and four under-expressed in response to *C. albicans*; LPS and *E. coli* each induced one overexpressed and two under-expressed analytes. These distinctions most likely reflect the different characteristics of the stimuli involved. Two of them, LPS and Pam3Cys, are defined biochemical compounds that interact with specific pattern recognition receptors: TLR4 and TLR2/1 heterodimer, respectively. *C. albicans, S. aureus*, and *E. coli* are all HK preparations of microorganisms containing complex mixtures of pathogen-associated molecular patterns that will interact with an array of different pathogen recognition receptors or other innate antigen receptors. It has been suggested that different microorganisms act on multiple, distinct TLRs simultaneously in a manner likened to the playing of a unique “chord” on a “molecular piano” (17) and the different response profiles generated by different stimuli described here may be an illustration of that effect.

The pathways by which BCG mediates its training effect *in vivo* are yet to be fully elucidated, although a role for the mycobacterial cell wall component muramyl dipeptide, acting *via* the NOD2 receptor, has been described (24). β-glucan, another activator of trained immunity mediates its effects *via* a dectin-1/Raf1-dependant pathway (33), yet the role of dectin-1 in recognizing mycobacteria is probably minor at most. The emerging picture is one in which the recall of a trained immune response may be restimulated *via* different receptors to those that prime that same response and that the cytokine/chemokine signature of that recall is determined by the receptors involved. Although little is known at this time about the mechanisms responsible, this should be a focus of future studies.

Despite the differences described above, we described here a biosignature of BCG-induced trained innate immunity in infants comprising increases in EGF, IL-6, and PDGF-AB/BB, all of which were apparent in responses to three different innate stimuli. This is different to the characteristic signature of trained innate immunity in adults, which includes TNFα and IL-1β as well as IL-6. It must be however underlined that in adults not all the cytokines reported here were measured, and some of them may be upregulated as well in older individuals. The absence of canonical innate cytokines such as TNFα in this infant biosignature may reflect the maturation of the infant innate immune response, whereby the ability to make certain cytokines develop over the first 9 months of life (34). Despite the most extensive response profile, *Mtb* lystae stimulation did not upregulate EGF or PDGF-AB/BB. This may reflect the involvement of a broader array of pattern recognition receptors in response to *Mtb* lystae with downstream effects that manifest themselves as a distinct pattern of upregulated and downregulated markers. These observations illustrate the importance of an unbiased, multiplex approach to analyzing these responses. A more directed study focused on TNFα, IL-6, and IL-1β would have underestimated the effect of trained immunity in infants and would have missed the previously unreported markers EGF and PDGF-AB/BB, as well as the finding that *C. albicans*-induced trained immunity recall in BCG-vaccinated infants involves reduced IL-2, IL-13, IL-17, and IP-10 responses. It is interesting that IL-17 responses to *C. albicans* are reduced in vaccinated infants. Th-17 cells are known to be important in immune responses to *C. albicans* (35). BCG vaccination has been shown to enhance non-specific (innate-mediated) protection against candidiasis in SCID mice (24) and adult BCG vaccination activates heterologous Th-17 responses specific for non-mycobacterial ligands including *C. albicans* (31). Our data suggest that BCG is exerting a different influence on the infant immune response, steering it away from a bias toward Th-17 development that is known to exist in early life (36). Detailed analyses of epigenetic and transcriptional programs of training or tolerance induction have revealed upregulation and downregulation of genes that characterize these responses hence it is unsurprising that the profiles we report here involve both upregulation and downregulation of different cytokines and chemokines associated with both T-cell and inflammatory monocyte responses to stimuli such as *C. albicans* as well as to *E. coli* and LPS which have both been associated with the induction of tolerance (37, 38).

In contrast to our findings here, another report of infant BCG-associated, non-specific cytokine responses described increases in TNFα and IL-1β as well as in IL-6 in response to Pam3Cys (39). Differences between study designs that might explain this difference are that Jensen et al. carried out their study in a low-income country and in low birth weight infants. In addition, the genetic backgrounds of the populations studied were different. Their sampling time point of 4 weeks was earlier than that used here and there were some methodological differences in their whole blood assay. An interesting possible explanation is that, unlike Jensen et al., infants in our study had received DTP vaccination through the national vaccination program by the time of sampling which is thought to negatively affect the positive non-specific effects of BCG (40). Finally, compared to Jensen et al., our study was relatively small and exploratory and was possibly underpowered to detect small differences in cytokine responses. It should be noted that in agreement with our findings and those of others (7), Jensen et al. found no BCG-associated increase in TNFα in response to non-specific LPS stimulation.

The involvement of monocytes in adult trained immunity has been demonstrated using stimulating ligands that interact with receptors known to be found on monocytes and more directly by the demonstration that markers of monocyte activation and differentiation are upregulated following training (24). It is also possible to isolate adult blood monocytes and train them directly with BCG and β-glucan *in vitro* (32). Although the assay described in this paper was designed primarily to look at secreted cytokines and chemokines, it was possible to harvest PBLs and to examine monocytes and NK cells for evidence of BCG vaccination-associated trained innate activation. We examined four markers of monocyte activation, but found no significant differences in their expression following non-specific stimulation of samples from vaccinated and unvaccinated infants. Stimulation with *Mtb* lystae, which also induced increases in 27 cytokines and chemokines in vaccinated infants, did increase levels of CD11b and CD207 on the surface of monocytes. Unlike responses induced by non-specific ligands in these experiments, it is likely that antigen-specific T-cell help is involved in these changes in monocyte activation. It is interesting that monocytes from BCG-vaccinated infants show increased expression of CD207 (mannose receptor), as this is usually found only on certain populations of differentiated macrophages and dendritic cells (41).

Natural killer cells from BCG-vaccinated infants displayed increased levels of the activation marker CD69 in response to Pam3Cys, as well as to *Mtb* lystae. Previous work has described long-lived mycobacteria-specific NK cells following infant BCG as well as a role for NK cells in BCG-induced trained immunity in adults and in BCG-vaccinated SCID mice which show increased resistance to *C. albicans* infection (25, 42). However, this is the first time their involvement in trained immunity to heterologous stimuli in infants has been described. NK cells express the TLR2 receptor *via* which they are reported to interact with mycobacteria (43, 44). NK cells are also activated in response to type 1 cytokines (45). Although we cannot determine the pathway of NK cell activation from the current data, we did observe a correlation between CD69 expression, IL-12p40 secretion, and IL-10 secretion in response to Pam3Cys. IL-12 is known to be a soluble activator of NK cells, while a role for IL-10 is less obvious. A limitation of this study was the small volume of blood available from infants which meant it was not possible to identify the cell type producing the cytokines and chemokines described. Clearly it would be interesting to determine whether the NK responses described above or other populations were a source, either by depletion studies or intracellular cytokine staining and future studies should address this. Additionally, as well as stimulation of the TLR2/1 heterodimer using Pam3Cys, it would be useful in future to determine more precisely the role of TLR2 either in homodimeric form or as a heterodimer with TLR6 also using specific ligands for these receptors.

This exploratory study was not designed to investigate the heterologous effects of infant BCG vaccination on clinical outcomes; however, if the non-specific immune responses we describe are to provide a mechanism for these effects, a causal relationship will need to be demonstrated in a larger study. The setting of such a study will need to be carefully considered as to date, the results of clinical trials have been mixed. Heterologous, BCG-induced protection against clinical outcomes related to infectious disease has been observed in a low-income setting in West Africa (15, 46) but was not recapitulated in another trial in a high income, European setting (47, 48).

In conclusion, we have described a novel whole blood signature of BCG-induced, trained innate immunity in infants that includes secretion of EGF, IL-6, and PDGF-AB/BB and involves activated NK cells. We also show distinct patterns of cytokine and chemokine release in response to different innate ligands. The data show different patterns to previously published descriptions of BCG-induced trained innate immunity in adults and low birth weight infants and suggest that more, larger scale studies of this effect in different populations are required for a more complete understanding.

## Ethics Statement

This study was carried out in accordance with the recommendations of the National Research Ethics Service Committee London-East and the ethics committee of the London School of Hygiene and Tropical Medicine with written informed consent from all subjects. All subjects gave written informed consent in accordance with the Declaration of Helsinki. The protocol was approved by the National Research Ethics Service Committee London-East and the ethics committee of the London School of Hygiene and Tropical Medicine.

## Author Contributions

SS performed the experiments, was responsible for the analysis and interpretation of data, and wrote the manuscript. JK and MN produced the panel of stimuli used in experiments. SS, JK, MN, and HD all contributed to the conception and design of the work, critically read and contributed to revisions of the manuscript, approved the final version of the manuscript, and agreed to be accountable for all aspects of the work including its accuracy and integrity.

## Conflict of Interest Statement

The authors declare that the research was conducted in the absence of any commercial or financial relationships that could be construed as a potential conflict of interest.
