# Supplementary material for: Whole Blood Profiling of Bacillus Calmette–Guérin-Induced Trained Innate Immunity in Infants Identifies Epidermal Growth Factor, IL-6, Platelet-Derived Growth Factor-AB/BB, and Natural Killer Cell Activation
Source: Front Immunol. 2017 Jun 6;8:644. doi: 10.3389/fimmu.2017.00644 (PMC5459878; doi:10.3389/fimmu.2017.00644)
Supplement: Supplementary file 1 [file Presentation_1.PDF]

Supplementary Figure 1

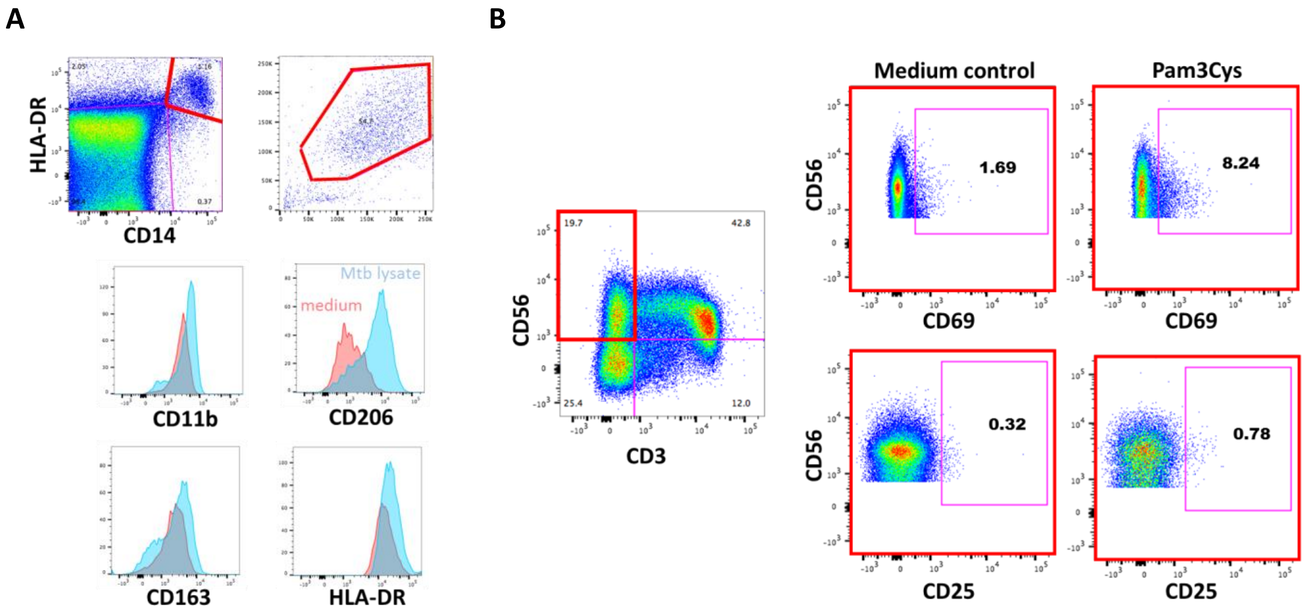

Figure S1. Gating strategy for monocyte and NK cell responses.
(A) Monocytes were gating sequentially on HLA-DR^{high}CD14^{high} expression and size and granularity. Activation marker expression on stimulated or control monocytes was determined by mean fluorescence intensity. (B) NK cells were gated on the basis of a CD56^{+}CD3^{-} profile. Activation marker expression on monocytes was determined by percent of NK cells positive for each marker. For both monocytes and NK cells, phenotype and activation marker gates were positioned based on staining profiles in fluorescence minus one control samples.
