# Supplementary material for: Whole Blood Profiling of Bacillus Calmette–Guérin-Induced Trained Innate Immunity in Infants Identifies Epidermal Growth Factor, IL-6, Platelet-Derived Growth Factor-AB/BB, and Natural Killer Cell Activation
Source: Front Immunol. 2017 Jun 6;8:644. doi: 10.3389/fimmu.2017.00644 (PMC5459878; doi:10.3389/fimmu.2017.00644)
Supplement: Supplementary file 2 [file Table_2.DOCX]

**Supplementary Table.** Concentrations (pg/ml) of cytokines/chemokines (median, 25^th^ percentile (LQ) and 75^th^ percentile (UQ)) measured by in unstimulated samples from BCG unvaccinated and BCG vaccinated infants

|  | BCG unvaccinated | | | BCG vaccinated | | |
| --- | --- | --- | --- | --- | --- | --- |
|  | Median | LQ | UQ | Median | LQ | UQ |
| **EGF** | 56.2 | 42.6 | 60.6 | 34.8 | 29.0 | 63.5 |
| **Eotaxin** | 41.9 | 22.6 | 55.9 | 27.5 | 23.2 | 65.1 |
| **FGF_2** | 22.2 | 3.2 | 57.8 | 20.8 | 3.2 | 55.7 |
| **Flt_3L** | 4.6 | 3.2 | 9.3 | 3.2 | 3.2 | 7.5 |
| **Fractalkine** | 55.1 | 3.2 | 80.2 | 42.1 | 34.0 | 71.4 |
| **G_CSF** | 6.9 | 3.2 | 31.1 | 3.2 | 3.2 | 8.6 |
| **GM_CSF** | 11.1 | 4.7 | 15.2 | 7.7 | 3.8 | 10.1 |
| **GRO** | 339.6 | 251.8 | 548.8 | 1512.6 | 938.2 | 1576.6 |
| **IFNa2** | 21.1 | 15.6 | 28.0 | 16.3 | 7.7 | 18.9 |
| **IFNg** | 4.5 | 3.2 | 7.3 | 3.3 | 3.2 | 4.0 |
| **IL_1a** | 3.4 | 3.2 | 4.6 | 3.2 | 3.2 | 3.2 |
| **IL_1b** | 3.2 | 3.2 | 3.2 | 3.2 | 3.2 | 3.2 |
| **IL_1Ra** | 15.7 | 11.5 | 31.4 | 11.7 | 7.8 | 14.1 |
| **IL_2** | 3.2 | 3.2 | 3.2 | 3.2 | 3.2 | 3.2 |
| **IL_3** | 3.2 | 3.2 | 3.2 | 3.2 | 3.2 | 3.2 |
| **IL_4** | 3.2 | 3.2 | 3.2 | 3.2 | 3.2 | 3.2 |
| **IL_5** | 3.2 | 3.2 | 3.2 | 3.2 | 3.2 | 3.2 |
| **IL_6** | 3.2 | 3.2 | 3.8 | 3.2 | 3.2 | 4.9 |
| **IL_7** | 3.3 | 3.2 | 3.6 | 3.2 | 3.2 | 3.5 |
| **IL_8** | 199.3 | 73.7 | 614.1 | 150.1 | 101.6 | 250.9 |
| **IL_9** | 3.2 | 3.2 | 3.2 | 3.2 | 3.2 | 3.2 |
| **IL_10** | 4.7 | 3.4 | 7.2 | 4.5 | 3.2 | 5.4 |
| **IL_12p40** | 13.5 | 11.7 | 18.8 | 9.5 | 5.2 | 13.2 |
| **IL_12p70** | 3.2 | 3.2 | 3.2 | 3.2 | 3.2 | 3.2 |
| **IL_13** | 3.2 | 3.2 | 3.2 | 3.2 | 3.2 | 3.2 |
| **IL_15** | 3.2 | 3.2 | 3.2 | 3.2 | 3.2 | 3.2 |
| **IL_17** | 3.2 | 3.2 | 3.2 | 3.2 | 3.2 | 3.2 |
| **IP_10** | 123.2 | 66.9 | 282.0 | 134.2 | 101.4 | 185.2 |
| **MCP_1** | 939.8 | 754.5 | 3278.0 | 1813.1 | 908.8 | 10000.0 |
| **MCP_3** | 118.5 | 31.7 | 198.8 | 78.0 | 35.6 | 185.5 |
| **MDC** | 219.2 | 147.1 | 348.5 | 268.1 | 196.9 | 333.8 |
| **MIP_1a** | 11.0 | 4.1 | 13.0 | 3.2 | 3.2 | 5.6 |
| **MIP_1b** | 56.2 | 40.5 | 69.7 | 46.3 | 33.2 | 60.7 |
| **PDGF_AA** | 583.9 | 572.7 | 1149.9 | 523.6 | 464.2 | 573.3 |
| **PDGF_ABBB** | 1548.5 | 962.2 | 2251.6 | 1211.2 | 826.5 | 1886.7 |
| **RANTES** | 2159.9 | 1615.5 | 2313.6 | 1649.6 | 1102.5 | 2165.1 |
| **sCD40L** | 69.2 | 58.9 | 79.6 | 71.0 | 43.5 | 100.0 |
| **sIL_2Ra** | 108.4 | 93.6 | 123.6 | 65.9 | 49.1 | 92.0 |
| **TGFa** | 3.2 | 3.2 | 3.2 | 3.2 | 3.2 | 3.2 |
| **TNFa** | 8.2 | 6.5 | 13.1 | 6.8 | 4.6 | 7.9 |
| **TNFb** | 3.2 | 3.2 | 3.6 | 3.2 | 3.2 | 3.2 |
| **VEGF** | 36.3 | 19.7 | 76.4 | 22.7 | 3.2 | 34.4 |
